# Hurricane Disturbance Stimulated Nitrification and Altered Ammonia Oxidizer Community Structure in Lake Okeechobee and St. Lucie Estuary (Florida)

**DOI:** 10.3389/fmicb.2020.01541

**Published:** 2020-07-10

**Authors:** Justyna J. Hampel, Mark J. McCarthy, Sanni L. Aalto, Silvia E. Newell

**Affiliations:** ^1^School of Ocean Science and Engineering, The University of Southern Mississippi, Ocean Springs, MS, United States; ^2^Department of Earth and Environmental Sciences, Wright State University, Dayton, OH, United States; ^3^Section for Aquaculture, The North Sea Research Centre, DTU Aqua, Technical University of Denmark, Hirtshals, Denmark

**Keywords:** AOA, AOB, eutrophication, harmful algal blooms, hurricane, nitrification

## Abstract

Nitrification is an important biological link between oxidized and reduced forms of nitrogen (N). The efficiency of nitrification plays a key role in mitigating excess N in eutrophic systems, including those with cyanobacterial harmful algal blooms (cyanoHABs), since it can be closely coupled with denitrification and removal of excess N. Recent work suggests that competition for ammonium (NH_4_^+^) between ammonia oxidizers and cyanoHABs can help determine microbial community structure. Nitrification rates and ammonia-oxidizing archaeal (AOA) and bacterial (AOB) community composition and gene abundances were quantified in Lake Okeechobee and St. Lucie Estuary in southern Florida (United States). We sampled during cyanobacterial (*Microcystis*) blooms in July 2016 and August 2017 (2 weeks before Hurricane Irma) and 10 days after Hurricane Irma made landfall. Nitrification rates were low during cyanobacterial blooms in Lake Okeechobee and St. Lucie Estuary, while low bloom conditions in St. Lucie Estuary coincided with greater nitrification rates. Nitrification rates in the lake were correlated (*R*^2^ = 0.94; *p* = 0.006) with AOA *amoA* abundance. Following the hurricane, nitrification rates increased by an order of magnitude, suggesting that nitrifiers outcompeted cyanobacteria for NH_4_^+^ under turbid, poor light conditions. After Irma, AOA and AOB abundances increased in St. Lucie Estuary, while only AOB increased in Lake Okeechobee. AOA sequences clustered into three major lineages: *Nitrosopumilales* (NP), *Nitrososphaerales* (NS), and *Nitrosotaleales* (NT). Many of the lake OTUs placed within the uncultured and uncharacterized NS δ and NT β clades, suggesting that these taxa are ecologically important along this eutrophic, lacustrine to estuarine continuum. After the hurricane, the AOA community shifted toward dominance by freshwater clades in St. Lucie Estuary and terrestrial genera in Lake Okeechobee, likely due to high rainfall and subsequent increased turbidity and freshwater loading from the lake into the estuary. AOB community structure was not affected by the disturbance. AOA communities were consistently more diverse than AOB, despite fewer sequences recovered, including new, unclassified, eutrophic ecotypes, suggesting a wider ecological biogeography than the oligotrophic niche originally posited. These results and other recent reports contradict the early hypothesis that AOB dominate ammonia oxidation in high-nutrient or terrestrial-influenced systems.

## Introduction

Canonical nitrification is a two-step process in which ammonium (NH_4_^+^) is oxidized to nitrite (NO_2_^–^), and NO_2_^–^ is further oxidized to nitrate (NO_3_^–^). Nitrification is a key pathway in the nitrogen (N) cycle as the sole biological link between oxidized and chemically reduced N forms, and it is often closely coupled to removal of excess N via denitrification (An and Joye, 2001). Thus, nitrification is a critical link in the ability of eutrophic ecosystems to mitigate excess N loads via coupling with denitrification.

The first step of nitrification, ammonia oxidation, is performed by ammonia-oxidizing archaea (AOA) and bacteria (AOB). Although AOA and AOB coexist in the environment, they generally exhibit niche separation (Prosser and Nicol, 2012; Hink et al., 2018). Previous studies on nitrifier community structure have posited that trophic status of the ecosystem defines the dynamics between AOA and AOB, based mostly on differences in substrate affinity for NH_4_^+^ and half-saturation constants (K_m_; Martens-Habbena et al., 2009; Bristow et al., 2016). Traditionally, AOB were thought to be more abundant in nutrient rich waters and soils (Jia and Conrad, 2009; Verhamme et al., 2011; Hou et al., 2013), while AOA were assumed to be more abundant in the oligotrophic open ocean (Francis et al., 2005; Könneke et al., 2005; Newell et al., 2011; Beman et al., 2012). However, a comprehensive phylogenetic analysis of >33,000 archaeal *amoA* sequences showed that AOA are ubiquitous, widespread, and highly diverse, and current knowledge on the physiology of cultured AOA does not represent predominant clades in the environment (Alves et al., 2018). Recently, various AOA clades from *Nitrososphaerales*, *Nitrosotaleales*, and *Nitrosopumilales* orders have shown to be highly abundant in shallow, eutrophic waters affected by agriculture runoff and cyanobacterial blooms (Zeng et al., 2012; Damashek et al., 2015; Hampel et al., 2018; Li et al., 2018), and wastewater treatment plants (Alves et al., 2018), where depth distribution of AOA ecotypes may not be applicable, and NH_4_^+^ concentrations often exceed those in the oligotrophic ocean by orders of magnitude. Thus, some AOA clades likely inhabit a more eutrophic niche than previously determined in ocean water column studies (Santoro et al., 2010; Sintes et al., 2013), but more work is needed to understand active ecotypes in shallow, freshwater systems.

Environmental controls on nitrification have been studied extensively in marine and coastal ecosystems (Beman et al., 2012; Horak et al., 2013; Urakawa et al., 2014; Damashek et al., 2016; Santoro et al., 2017), while studies in freshwater systems (Hugoni et al., 2013; Vissers et al., 2013; Bollmann et al., 2014; Hayden and Beman, 2014), particularly eutrophic systems, have received less attention (Hampel et al., 2018) and are often limited to sediment studies (Hou et al., 2013; Yao et al., 2018). Work in marine and coastal environments has shown that oxygen, temperature, salinity, light, turbidity, top-down grazing pressure (Lavrentyev et al., 1997; Jochem et al., 2004), and ambient NH_4_^+^ concentrations can all influence nitrification rates (Heiss and Fulweiler, 2016; Damashek and Francis, 2018). However, in eutrophic lakes, competition for NH_4_^+^ between nitrifiers and photoautotrophs, including bloom-forming cyanobacteria (cyanoHABs), may be more important than ambient NH_4_^+^ concentrations (which can be very high). Nitrifiers and cyanobacteria are both highly competitive for NH_4_^+^ and urea, and while cyanobacteria can assimilate N_2_ and NO_3_^–^, nitrifiers cannot. Cyanobacteria can also thrive in high light conditions, which may inhibit nitrifiers (Hayden and Beman, 2014). A recent study showed that this competition can reduce nitrification efficiency (Hampel et al., 2018), potentially impacting the capacity for denitrification and removal of excess N.

We examined environmental controls on nitrification rates and ammonia oxidizer community structure along a freshwater-estuarine continuum during cyanoHABs and before and after passage of a major hurricane. Lake Okeechobee and the St. Lucie Estuary (Florida) provide an opportunity to investigate the impacts of dissolved inorganic N (DIN) concentrations, salinity, cyanoHABs, and physical disturbance (hurricane passage) on nitrification. Lake Okeechobee has experienced toxic cyanoHABs for decades (James et al., 2009), resulting in myriad negative effects on the downstream St. Lucie Estuary. To prevent flooding, nutrient-laden water from Lake Okeechobee is released into St. Lucie Estuary via a canal system, reducing salinity and increasing nutrient concentrations and turbidity (Lapointe et al., 2012; Phlips et al., 2012). Toxic *Microcystis* blooms in St. Lucie Estuary have been observed on numerous occasions (Phlips et al., 2012), including during a massive bloom in 2016, which led the State of Florida to declare a state of emergency (Kramer et al., 2018).

On September 10th, 2017, Hurricane Irma made landfall in the Florida Keys as a Category 4 hurricane (sustained winds >210 km h^–1^) and moved through central Florida with sustained winds of ∼65 km h^–1^ in the Okeechobee region (Hampel et al., 2019) and maximum winds up to ∼100 km h^–1^ (SFWMD). High winds and precipitation runoff have major impacts on hydrology, microbial and phytoplankton community structure, sediments, and nutrients, including reducing salinity in estuaries, often leading to stratification and bottom-water hypoxia (Paerl et al., 2001). Hurricane winds also produce strong currents and seiches, which resuspend sediments and increase total suspended solids (TSS) and turbidity in the water column (James et al., 2008). Short-term hurricane effects on cyanoHABs include decreased NH_4_^+^ uptake rates and microcystin synthetase gene abundance (Hampel et al., 2019). Previous studies showed decreased AOA abundance and diversity after an oil spill (Newell et al., 2014) and increased AOB abundance after storm disturbance (Happel et al., 2018). However, very little is known about storm effects on nitrification and ammonia oxidizer community structure in freshwater and estuary ecosystems (Bouskill et al., 2011; Newell et al., 2014; Happel et al., 2018).

Despite its ecological importance, nitrification in eutrophic, cyanoHABs-dominated systems remains understudied. The goal of this study was to quantify nitrification rates and *amoA* gene abundance, and investigate ammonia oxidizer community structure, along a freshwater-estuarine continuum in central Florida during cyanoHABs in 2016 and 2017 and before and after Hurricane Irma passage. We hypothesized that nitrification rates would be lower during cyanoHABs due to resource competition and that hurricane disturbance would further decrease nitrification rates and the abundance of ammonia oxidizers. We also speculated that there would be a shift in *amoA* community structure as an immediate response to hurricane disturbance. Climate change predictions forecast escalation of eutrophication, cyanoHABs, and the intensity of large-scale extreme weather events (Paerl et al., 2019). Thus, understanding the effects of these disturbances on nitrification and ammonia oxidizer communities is important for constructing and validating ecosystem models used to evaluate ecosystem resilience and inform management action in eutrophic waters.

## Materials and Methods

### Sampling

**FIGURE 1:**
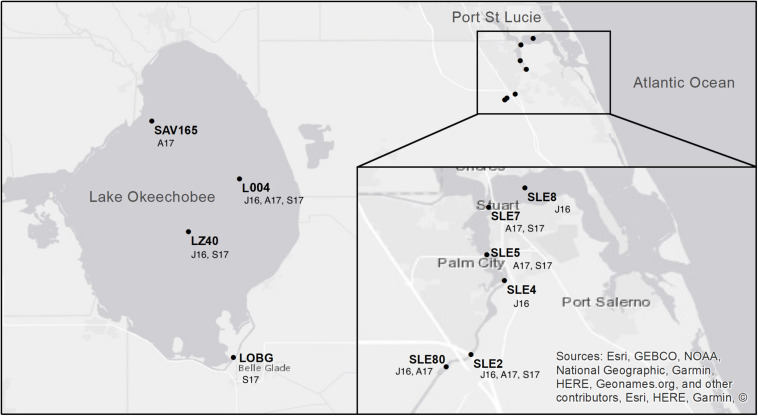
Map of sampling locations in Lake Okeechobee and St. Lucie Estuary. Annotations indicate sampling date (J16, July 2016; A17, August 2017; S17, September 2017). Modified from Hampel et al. (2019).

Lake and estuary water samples were collected on July 25–27, 2016, August 22–24, 2017, and September 20–21, 2017. The July 2016 sampling occurred during a large cyanoHAB in both systems (Kramer et al., 2018). In August 2017, sampling occurred 2 weeks before Hurricane Irma passed over the Okeechobee region as a Category 3 hurricane. The September 2017 sampling occurred 10 days after Hurricane Irma passed through south and central Florida. In July 2016, sampling was conducted at two stations in Lake Okeechobee, L004 (surface and bottom water) and LZ40 (surface and bottom water), and four stations in the St. Lucie Estuary following a freshwater to marine salinity gradient from SLE80, SLE2, SLE4, and SLE8 (surface water only; Figure 1). In August 2017, L004 (surface and bottom water) and a northern lake station (SAV165, surface water only) were sampled, along with SLE80, SLE5, and SLE7 in the estuary. Post-hurricane sampling was hindered by flooding and included stations L004, LZ40, and LOBG (nearshore in the south) in the lake (surface water only), and SLE5 and SLE7 in the estuary.

Water for nutrient analyses was filtered in the field to 0.2 μm with pre-rinsed syringe filters (Nylon) into 15 ml polypropylene tubes and frozen upon return to the laboratory. Water for nitrification experiments was collected with a van Dorn water sampler into 5 L cubitainers and returned to the lab for incubations. Physicochemical parameters were measured with a YSI multisensor sonde in July 2016 and August 2017, and a Manta 2 multiparameter sonde in September 2017. Dissolved nutrient analyses included NH_4_^+^, NO_2_^–^, NO_3_^–^, orthophosphate (ortho-P), and urea were analyzed using a Lachat Quickchem 8500 FIA nutrient analyzer. Turbidity and TSS measurements were obtained from the DB Hydro database^1^ and collected by South Florida Water Management District (SFWMD) according to standard EPA protocols.

### DNA Collection and Extraction

Environmental DNA was collected in August and September 2017. Samples were hand filtered in the field with 0.2 μm Sterivex filters (SVGP01015; MilliporeSigma, MA, United States). Samples were preserved in the field with ∼2 mL of Ambion RNAlater (Invitrogen, Carlsbad, CA, United States). The volume of site water pushed through Sterivex filters in August 2017 ranged from 120–240 ml. However, due to increased turbidity after the hurricane, only 45–60 ml of water was filtered in September for stations L004, LZ40, SLE5, and SLE7. An exception was station LOBG, where 300 ml of water were filtered. Preserved filters were frozen at −80°C until analysis.

Filter cartridges were thawed on ice in the lab, and RNAlater in the Sterivex filters was removed by pushing 10 ml of Phosphate Buffer Saline 1× Solution (Fisher BioReagents, United States) through the filter. DNA was extracted using the Gentra PureGene kit (Qiagen Inc., United States). Lysis buffer (0.9 ml) and Proteinase K (10 μl) were added to the filters, and the samples were incubated for 1 h at 55°C and 1 h at 65°C, repeated twice, in a hybridization oven at 90 rpm (Newell et al., 2011; Hampel et al., 2018).

### *amoA* Gene Abundance

Quality and quantity of the extracted DNA were measured spectrophotometrically (NanoDrop 2000, Thermo Scientific). There are limitations to using the NanoDrop at low DNA concentrations, but our samples had consistently high concentrations (>10 ng/μL). Archaeal *amoA* was quantified with Arch-amoAF and Arch-amoAR primers, and bacterial *amoA* was quantified with amoAF and amoA2R primers (Supplementary Table S4; Rotthauwe et al., 1997; Francis et al., 2005). Archaeal and bacterial *amoA* qPCR standards were prepared by cloning the fragment of interest with the TOPO TA Cloning Kit (Invitrogen, United States), inserting it into a competent cell plasmid (One Shot *E. coli* cells, Invitrogen, United States), and isolating the plasmid containing the *amoA* gene using the UltraClean Standard Mini Plasmid Prep Kit (Mo Bio Laboratories Inc., Carlsbad, CA, United States). Each qPCR run included three no template controls (NTC), six standards from serial dilution in triplicate, and the environmental DNA samples in triplicate. Each sample and standard received 10 μl of Luna Universal qPCR Master Mix (New England Biolabs Inc., United States), 0.5–1 μl of each 10 μM primer, and 20–35 ng of DNA template.

*amoA* qPCR protocols followed methods of Bollmann et al.(2014; and Supplementary Table S4), followed by melting curve analysis to ensure a single product. Automatic settings for the thermocycler (Realplex, Eppendorf) were used to determine threshold cycle (Ct values), efficiency (93–98%), and a standard curve with *R*^2^ values ≥0.99. Gene copy number was calculated as (ng ^∗^ number mol ^–1^)/(bp ^∗^ ng g^–1^^∗^ g mol^–1^ of bp) and is reported in *amoA* gene copies ml^–1^ of sample water.

### *amoA* Amplicon Sequencing and Analysis

Barcoded amplicons for AOA and AOB were prepared by using a two-step PCR approach (Herbold et al., 2015). In the first PCR, bacterial and archaeal *amoA* fragments were amplified using the standard procedures and primers listed above, with a decreased number of cycles (24 cycles). In the second PCR, the first-step PCR product was used as a template for eight cycle amplification with a primer consisting of the barcodes and Illumina adapters (Supplementary Table S4). The barcoded amplicons were sequenced on an Illumina MiSeq for paired end sequencing (2 × 300 bp PE). Barcoding, cleaning, quantification, and pooling was conducted at the Ohio State Molecular and Cellular Imaging Center, and the Illumina MiSeq Reagent kit v3, paired end 300 sequencing kit, and the Illumina Nextera XT index kit v2 for indexing the libraries were used. Raw sequence data analysis was conducted using Mothur pipeline (version 1.42.3; Schloss et al., 2009). Sequences were assembled into contigs, and adapters and primers were removed. Sequences longer than 555 bp for AOA, and 475 bp for AOB, and low-quality sequences with more than one mismatch in barcode/primer sequences, were removed in Mothur. Frameshift errors in unique archaeal and bacterial reads were corrected using the FunGene FrameBot tool (Wang et al., 2013), and sequence alignment was conducted using aligned archaeal or bacterial *amoA* sequences retrieved from the FunGene database (Aalto et al., 2018). Chimeric sequences were removed using Uchime in Mothur (Edgar et al., 2011), and sequences were clustered into OTUs at 95% sequence identity.

The taxonomic assignment of archaeal and bacterial sequences was completed in Mothur using the Alves et al. (2018) comprehensive AOA *amoA* database and custom AOB *amoA* reference libraries, which were collected from sequences and their taxonomies in the Nucleotide database (Aalto et al., 2018) at a 95% cutoff (Beman and Francis, 2006; Frank et al., 2016; Aalto et al., 2018). Phylogenetic analysis was conducted in MEGA X, where sequences were aligned using the multiple sequence alignment tool, ClustalW, to align representative and reference sequences. Maximum likelihood phylogenetic trees were constructed with 1000 bootstrap replicates using the Tamura-Nei nucleotide substitution model in MEGA X (Kumar et al., 2018). The phylogenetic tree was edited and annotated using iTOL v.5 (Letunic and Bork, 2019). Raw sequences were submitted to NCBI’s Sequence Read Archive under BioProject #PRJNA592084.

### Nitrification Rates

Nitrification rates were measured using the ^15^NH_4_^+^ tracer addition method (Ward, 2008; Newell et al., 2013; Heiss and Fulweiler, 2016). Five hundred ml of water from each station was distributed into acid-washed, 1 L polycarbonate bottles and enriched with 98% ^15^NH_4_Cl (Isotec) at final concentrations of 0.25 μM (2016) or 0.5 μM (2017). Amended water was mixed thoroughly by inverting 10 times and distributed into three, acid-washed 125 ml polycarbonate incubation bottles. Unamended control samples for each station were distributed into 125 ml incubation bottles. Initial samples (T_0_) were filtered to 0.22 μm with syringe filters into 50 ml polycarbonate tubes and frozen until analysis, and final samples (T_*f*_) were collected after incubating for 20 h at near *in situ* light and temperature in a laboratory incubator.

Accumulation of ^15^NO_3_^–^ was measured using the Cd reduction/NaN_3_ reduction method (McIlvin and Altabet, 2005; Heiss and Fulweiler, 2016). Approximately 25 ml from each sample was filtered (0.2 μm syringe filter) into 50 ml centrifuge tubes. NO_3_^–^ was reduced to NO_2_^–^ by addition of 100 mg of MgO, 6.6 g of NaCl, and 0.75–1 g of acidified Cd powder to each sample, followed by a 17 h incubation on a shaker table (McIlvin and Altabet, 2005). Samples were centrifuged at 1000 × *g* for 15 min, and 7.5 ml of supernatant was carefully transferred into 12 ml Exetainers.

NOx (including cadmium-reduced NO_2_^–^) was further reduced to N_2_O with the NaN_3_ method (McIlvin and Altabet, 2005; Newell et al., 2011; Hampel et al., 2018). Briefly, each sample was treated (with gastight syringe) with 0.25 ml of a 1:1 (v:v) solution of 2 M NaN_3_:20% CH_3_COOH solution (Ar-purged) and incubated for 1 h at 30°C (McIlvin and Altabet, 2005). NO_2_^–^ in the sample from Cd reduction was transformed chemically to N_2_O. After a 1 h incubation, the reaction was stopped with an injection of 0.15 ml of 10 M NaOH. Samples were inverted and sent to the University of California Davis Stable Isotope Facility for isotopic analysis of ^45/44^N_2_O using a Thermo Finnigan GasBench + PreCon trace gas concentration system interfaced to a Thermo Scientific Delta V Plus isotope-ratio mass spectrometer (Bremen, Germany). Nitrification rates were corrected for NaN_3_ reduction efficiency (determined from reduced standards), and ^15^NO_3_^–^ production was calculated as:

Nitrification(innmolLd-1)-1=((N15/N*14[NO]3-)-24h(N15/N*14[NO]3-))0h/α*t

Where α = [^15^NH_4_^+^]/([^15^NH_4_^+^] + [^14^NH_4_^+^]).

### Statistical Analysis

Statistical analysis was conducted using RStudio software (Version 1.1.463). First, environmental data, nitrification rates, and *amoA* abundance were checked for normality using the Shapiro-Wilk test. After determining that the data were not normally distributed, the Kendall correlation method for non-parametric data was used to determine correlations between parameters. Wilcoxon and Kruskal-Wallis tests for non-parametric data were used to determine differences between sampling events. Additionally, a stepwise multiple-regression model for nitrification rates was constructed using the MASS package (R Version 7.3). The best-fitting model was selected based on the minimum Akaike’s information criteria (AIC; Akaike, 1987). All non-normally distributed variables were log(x + 1)-transformed prior to running the model to normalize the data for parametric analysis.

Community structure analysis and visualization was performed using phyloseq (Version 1.28.0; McMurdie and Holmes, 2013), vegan (Version 2.5-2; Oksanen et al., 2018), and ggplot2 (Version 3.2.0; Wickham, 2016) packages in RStudio. Alpha diversity was calculated for bacterial and archaeal data. Bray-Curtis dissimilarity analysis was used to evaluate variability of communities across samples, using non-metric multidimensional scaling (NMDS; Supplementary Figure S1). Differences in the AOA and AOB community structure between sampling events were tested separately with permutational multivariate analysis of variance (PERMANOVA; Anderson, 2001; adonis in vegan package), followed by the homogeneity of dispersion test using the betadisper function. Relationships between sequencing data and environmental variables were evaluated using the Constrained Analysis of Principal Coordinates (CAP) ordinations after removing missing values from environmental data (Supplementary Figure S2).

## Results

### Physical and Biogeochemical Parameters

Environmental data have been reported previously (Hampel et al., 2019) and are presented here in Supplementary Tables S1, S2. Water temperature in the lake and estuary was uniform across all sampling events. After the hurricane, salinity and NH_4_^+^ concentrations decreased in the estuary (*p* = 0.03, *p* = 0.08, respectively), while conductivity (*p* = 0.005), turbidity (*p* = 0.001), TSS (*p* = 0.005), and NO_3_^–^ concentrations (*p* = 0.001) increased (Supplementary Tables S1, S2). Post-hurricane, lake turbidity (*p* = 0.04) and NO_3_^–^ concentration (*p* = 0.05) increased. In the lake, chlorophyll *a* (chl *a*) concentration was higher during the August 2017 bloom than during the 2016 bloom and decreased after the hurricane (Supplementary Table S1). In the estuary, chl *a* concentrations decreased along the salinity gradient and were lowest following the hurricane (Supplementary Table S2).

### Nitrification Rates

**FIGURE 2:**
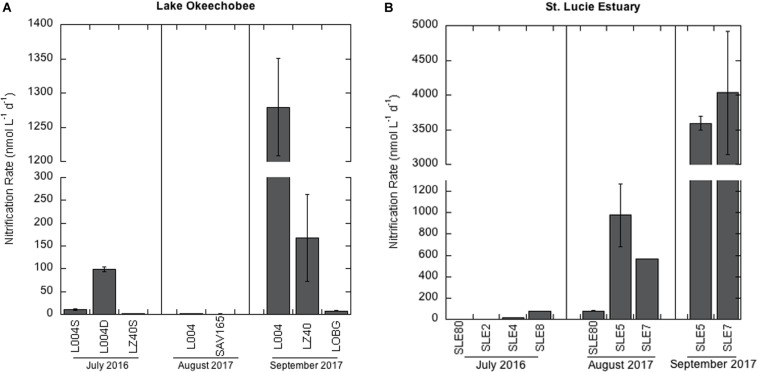
Nitrification rates ± one standard error in: **(A)** Lake Okeechobee and **(B)** St. Lucie Estuary. Note the scale difference between **(A)** and **(B)**.

Nitrification rates in the lake in July 2016 varied between the eastern (L004; Figure 1) and central lake stations (LZ40; Figure 2A). Nitrification rates were higher in bottom water (2 m) at L004 (98.8 ± 5.11 nmol L^–1^ d^–1^; mean ± SE) compared to surface water (∼0.2 m; 10.5 ± 1.6 nmol L^–1^ d^–1^; *p* = 0.03). In contrast, nitrification rates in the bottom water at LZ40 were undetectable, and low rates were measured in surface water (2.04 ± 0.52 nmol L^–1^ d^–1^). Nitrification rates in the lake in August 2017 were only measurable in surface water and were slightly lower than those measured in 2016 (0.78 ± 0.82 nmol L^–1^ d^–1^ at SAV165 to 2.14 ± 0.02 nmol L^–1^ d^–1^ at L004; Figure 2A). However, after the hurricane, nitrification rates increased by 2–3 orders of magnitude (*p* = 0.003) at L004 (1280 ± 71.3 nmol L^–1^ d^–1^) and LZ40 (168 ± 96.1 nmol L^–1^ d^–1^).

In July 2016, nitrification rates in the estuary increased along the salinity gradient, with undetectable rates at the most upstream site (SLE80) and greatest rates at the most saline site (SLE8; 79.4 ± 0.01 nmol L^–1^ d^–1^; Figure 2B). In August 2017, nitrification rates in the estuary were higher than in 2016 (*p* = 0.06). Again, the lowest rates were observed upstream near the St. Lucie canal (SLE80; 79.1 ± 2.22 nmol L^–1^ d^–1^), and greatest rates were observed downstream at SLE5 (978 ± 294 nmol L^–1^ d^–1^; *p* = 0.04) and SLE7 (563 ± 0.4 nmol L^–1^ d^–1^; *p* < 0.001; Figure 2B). Following the hurricane, nitrification rates increased at both upstream (SLE5: 3590 ± 98.9 nmol L^–1^ d^–1^) and downstream stations (SLE7: 4030 ± 888 nmol L^–1^ d^–1^; Figure 2B; *p* < 0.001). Overall, nitrification rates in the estuary exceeded those in the lake on all occasions. Nitrification rates were negatively correlated with temperature and dissolved oxygen (DO; Kendall, *p* = 0.03; Table 1) and positively correlated with ambient NO_3_^–^ concentrations (Kendall, *p* = 0.001). Additionally, the best-fitting multiple regression model for nitrification revealed that ambient NO_3_^–^ concentration, turbidity, and salinity were the best predictors for nitrification rates in the lake and estuary (Adj. *R*^2^ = 0.99; Supplementary Table S3).

**TABLE 1:** Details of Kendall correlation for non-parametric data analysis of environmental parameters, geochemical rates, and *amoA* gene abundance in Lake Okeechobee and St. Lucie Estuary.

		Temp.	DO	Salinity	NH_4_^+^	NO_3_^–^	Cond.	Turbidity	TSS
Nitrification	Kendall T	**−0.58**	**−0.38**	0.39	0.08	**0.58**	0.29	0.23	0.00
	*p*-value	**0.002**	**0.03**	0.09	0.65	**0.001**	0.110	0.45	1.00
AOA	Kendall T	**−0.51**	0.37	**−0.40**	**−0.73**	0.24	0.17	0.52	**0.68**
	*p*-value	**0.04**	0.12	0.32	**0.003**	0.32	0.47	0.09	**0.003**
AOB	Kendall T	**−0.04**	**−0.11**	**−0.60**	0.02	0.28	**0.53**	0.33	0.29
	*p*-value	0.85	0.65	0.14	0.92	0.24	**0.03**	0.29	0.36

*Correlations where *p* < 0.05 are presented in bold. Temp., temperature; DO, dissolved oxygen; Cond., conductivity; TSS, total suspended solids.*

### *amoA* Abundance

**FIGURE 3:**
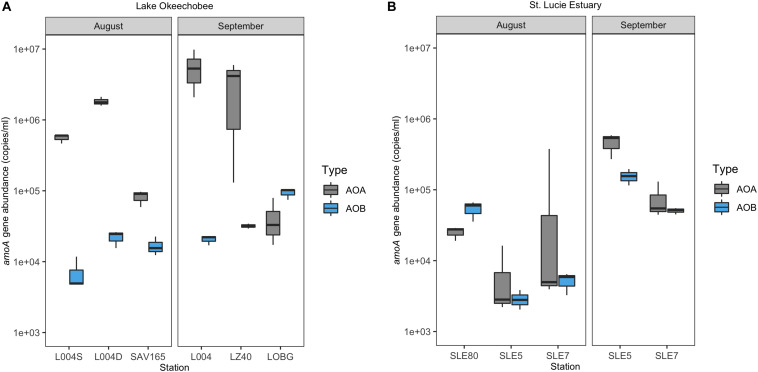
Ammonia oxidizer abundance (*amoA* gene) ± one standard error in Lake Okeechobee **(A)** and St. Lucie Estuary **(B)** before and after Hurricane Irma.

**FIGURE 4:**
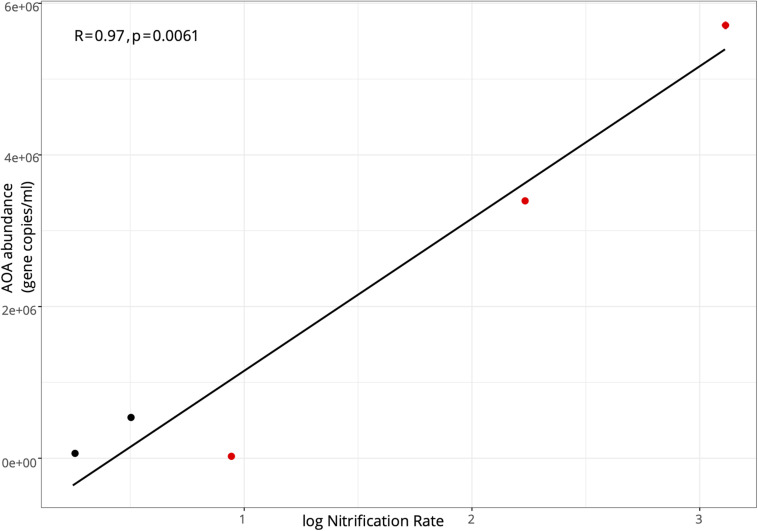
Pearson correlation between the abundance of ammonia oxidizing archaea *amoA* functional gene and log-transformed nitrification rates in Lake Okeechobee. Black points indicate samples collected in August 2017; red points indicate samples collected in September 2017.

In the lake in August 2017, AOA (mean = 8.21 ± 0.70 × 10^5^; range: 8.21 × 10^4^–1.83 × 10^6^) exceeded AOB abundance (mean = 1.54 ± 0.29 × 10^4^; range: 2.31–3.29 × 10^3^) at all stations, with AOA abundance 1–3 orders of magnitude higher (Figure 3A). Following the hurricane, AOA abundance (mean = 3.06 ± 1.32 × 10^6^; range: 4.32 × 10^4^–5.73 × 10^6^) was also higher than AOB (mean = 4.87 ± 0.43 × 10^4^; range: 2.07–9.35 × 10^4^) at all stations, except near the lake shore (LOBG; Figure 3A). AOB abundance increased after Hurricane Irma in the lake (*p* = 0.05), while AOA abundance did not change. Lake nitrification rates were correlated with AOA *amoA* copy numbers (Figure 4; *R*^2^ = 0.94, *p* = 0.006), while no pattern was observed between nitrification rates and lake AOB or estuarine ammonia oxidizers (Supplementary Figure S3).

In the estuary in August 2017, AOA abundance (mean = 5.36 ± 4.39 × 10^4^; range: 7.10 × 10^3^–1.29 × 10^5^) exceeded AOB (mean = 2.07 ± 0.36 × 10^4^; range: 5.25 × 10^2^–9.37 × 10^3^) at all stations except upstream (SLE80). AOA abundance was slightly lower than in the lake, while AOB abundances were comparable to those measured in the lake (Figure 3B). After Hurricane Irma, *amoA* gene copies for both AOA and AOB increased, and AOA (mean = 2.71 ± 0.63 × 10^5^; range: 7.65 × 10^4^–4.65 × 10^5^) outnumbered AOB (mean = 1.03 ± 0.13 × 10^5^; range: 5.06 × 10^4^–1.56 × 10^5^) at all stations (Figure 3B). AOA abundance in both the lake and estuary were negatively correlated with temperature and ambient NH_4_^+^ concentration and positively correlated with TSS (Kendall, *p* = 0.003; Table 1). AOB abundance was positively correlated with conductivity (Kendall, *p* = 0.03).

### Ammonia Oxidizer Community Structure

**FIGURE 5:**
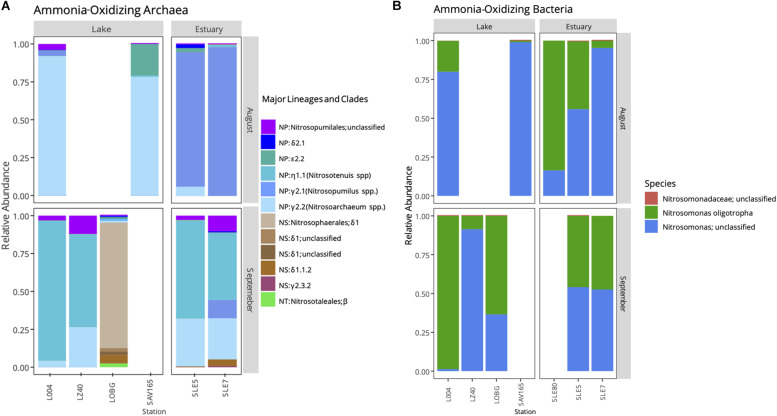
Relative abundance of ammonia-oxidizing archaea **(A)** and bacteria **(B)** in Lake Okeechobee and St. Lucie Estuary before (August) and after (September) hurricane disturbance. AOA represent three major lineages: *Nitrosopumilales* (NP), *Nitrososphaerales* (NS), and *Nitrosotaelales* (NT). Lineages are divided into clades (Greek letters) and numbers (genera).

Following the quality control steps in Mothur, frameshift in FrameBot, and chimera removal, we retrieved 3,699 unique *amoA* sequences for AOA and 17,030 for AOB. *amoA* sequences grouped into 574 and 101 OTUs (95% similarity) for AOA and AOB, respectively. We observed a shift in the AOA community after the hurricane (Figure 5; PERMANOVA, *F* = 2.763, *p* = 0.03), while the AOB community changed only marginally (*F* = 1.592, *p* = 0.08).

**FIGURE 6:**
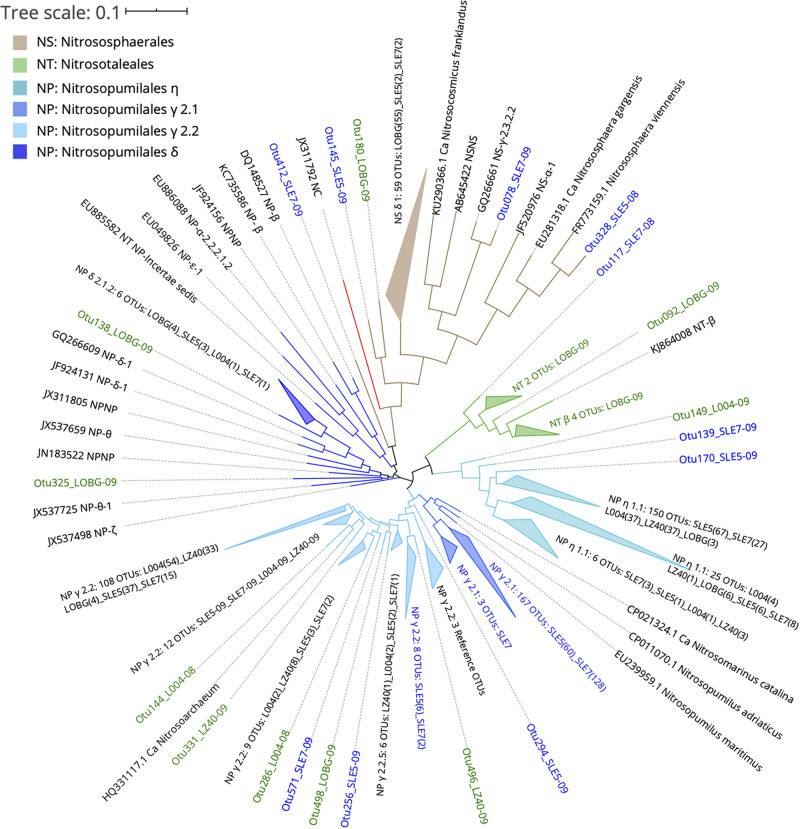
Phylogenetic tree of ammonia-oxidizing archaea OTUs classified from *amoA* reads at 95% similarity with reference sequences (Alves et al., 2018). OTU label colors indicate OTUs found in Lake Okeechobee (green), St. Lucie Estuary (blue), or both systems (black). Numbers in parenthesis indicate the number of OTUs present from each station. Nodes with branch length <0.05. Some nodes were further collapsed for clarity.

Ammonia-oxidizing archaeal sequences grouped phylogenetically into the lineages and clades of *Nitrososphaerales* (NS), *Nitrosotaleales* (NT), and *Nitrosopumilales* (NP): NP η (*Nitrosotenuis* spp.), NP γ 2.1 (*Nitrosopumilus* spp.), NP γ 2.2 (*Nitrosoarchaeum* spp.), and NP δ (Figure 6). Sequences from the lake and estuary were mostly phylogenetically distinct, as OTUs present only in the lake were phylogenetically close to NS and NT orders, while the estuarine-only OTUs assigned to the NP γ 2.1 clade.

Before the hurricane, AOA were dominated by unclassified NP γ 2.2 (*Nitrosoarchaeum* spp.) in the lake and NP γ 2,1 (*Nitrosopumilus* spp.) OTUs in the estuary (Figure 5A). After the hurricane, AOA in the lake shifted to NP η 1.1 (*Ca. Nitrosotenuis cloacae*) OTUs in the central lake (L004, LZ40), while most OTUs from the nearshore station (LOBG) clustered within the NS δ clade. The post-hurricane AOA community in the estuary shifted to higher relative abundances of NP η 1.1 (*Ca. N. cloacae*) upstream (SLE5) and NP η 1.1 and NP γ 2.2 OTUs downstream (SLE7).

**FIGURE 7:**
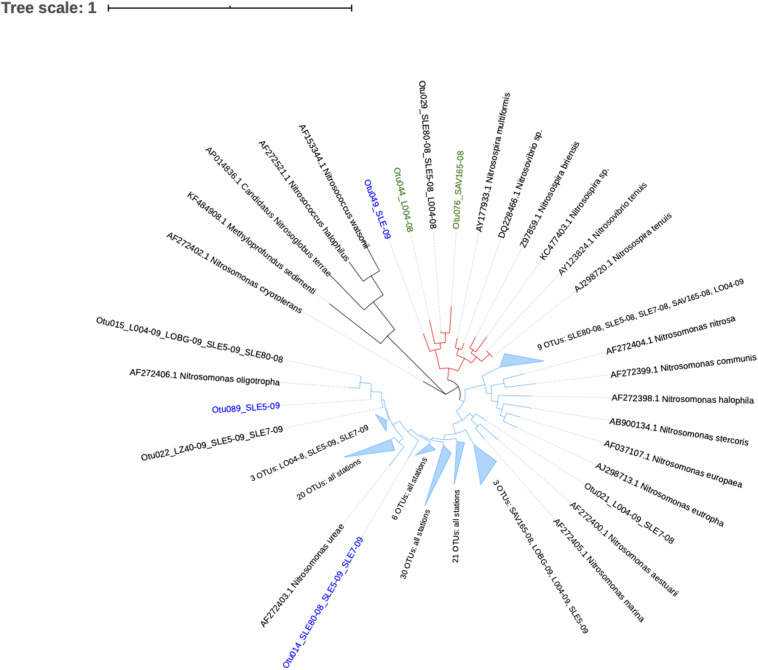
Phylogenetic tree of ammonia-oxidizing bacteria OTUs classified from *amoA* reads at 95% similarity. OTU label colors indicate OTUs found in Lake Okeechobee (green), St. Lucie Estuary (blue), or both systems (black). Numbers in parenthesis indicate the number of OTUs present from each station. Nodes with branch length <0.05. Some nodes were further collapsed for clarity.

Ammonia-oxidizing bacterial sequences were not as clearly separated by sampling location as AOA sequences and were placed phylogenetically into the *Nitrosomonas* clusters, with most of the OTUs close to *Nitrosomonas ureae*, and some estuary-only OTUs close to *Nitrosomonas nitrosa* (Figure 7). Before the hurricane, AOB communities were dominated by *Nitrosomonas oligotropha* in the upstream estuary (SLE80 and SLE5) and by unclassified *Nitrosomonas* groups in the lake (Figure 5B). After the hurricane, both lake and estuary AOB communities shifted to a higher proportion of *Nitrosomonas oligotropha.*

**FIGURE 8:**
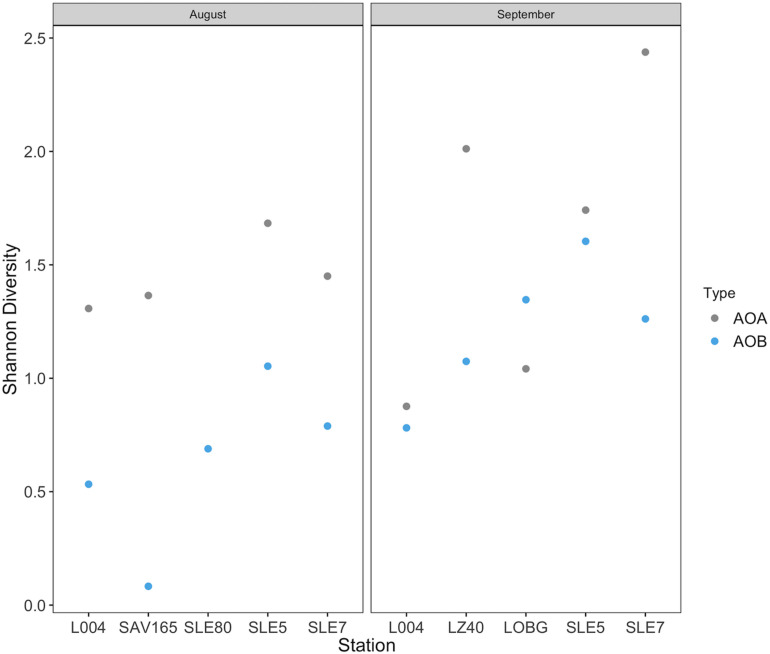
Shannon diversity before and after Hurricane Irma in Lake Okeechobee and St. Lucie Estuary for ammonia oxidizing archaea and ammonia oxidizing bacteria.

Overall, despite an order of magnitude fewer sequences, AOA were more diverse than AOB (*p* = 0.009; Figure 8). AOB alpha diversity increased after the hurricane in both systems (*p* = 0.02), while AOA diversity remained unchanged (Figure 8). Constrained Analysis of Principal coordinates (CAP) for AOA showed separation by sampling month and the effects of hurricane disturbance (Supplementary Figure S2A). CAP1 (57.9% variance explained) was driven by salinity (*r* = −0.95), NH_4_^+^ concentration (*r* = −0.91), and turbidity (*r* = 0.77), which were also affected by the hurricane, while CAP2 (25.9% variance explained) was driven primarily by NO_3_^–^ (*r* = −0.61) and DO concentrations (*r* = 0.53). For AOB communities (Supplementary Figure S2B), CAP1 (35.2% variance explained) was associated with salinity (*r* = 0.80), NH_4_^+^ concentration (*r* = 0.76), and nitrification rate (*r* = −0.63), while CAP2 (20.7% variance) was associated with salinity (*r* = 0.54) and turbidity (*r* = −0.51).

## Discussion

### Nitrification During Cyanobacterial Blooms

Nitrification rates in both the lake and estuary varied between sampling events (Figure 2). Nitrification rates in Lake Okeechobee were low during cyanoHABs in 2016 and 2017 (Figure 2A), similar in magnitude to those from oligotrophic Lake Superior (Small et al., 2013), and lower than those previously measured in spring in Lake Okeechobee (James et al., 2009). Competition for NH_4_^+^ between nitrifiers and photoautotrophs, including cyanobacteria, may inhibit nitrification during severe cyanoHABs, which were observed in Lake Okeechobee in 2016 (Kramer et al., 2018; Hampel et al., 2019). NH_4_^+^ is the most energetically favorable N form for primary producers, including non-N_2_ fixing cyanobacteria (e.g., *Microcystis*), which often outcompete other algal groups (e.g., Blomqvist et al., 1994) and ammonia oxidizers for NH_4_^+^. Nitrifiers have a lower substrate affinity and higher K_*m*_ (Martens-Habbena et al., 2009) for NH_4_^+^ than *Microcystis* (Nicklisch and Kohl, 1983; Baldia et al., 2007). In Lake Okeechobee, nitrification accounted for <1% of total NH_4_^+^ uptake and was likely suppressed by competition with *Microcystis* (Hampel et al., 2019). Suppression of nitrification due to competition for NH_4_^+^ between *Microcystis* and ammonia oxidizers has also been observed in hypereutrophic Lake Taihu (China), where nitrification rates during massive *Microcystis* blooms were equally as low as reported here in Lake Okeechobee, but increased when the bloom was less pronounced (Hampel et al., 2018).

Water column nitrification measurements in lakes are scarce compared to open ocean and coastal marine systems, but the rates measured here are comparable to other systems. In the estuary, nitrification rates in 2016 ranged from undetectable to 79.4 ± 0.02 nmol L^–1^ d^–1^ and increased along the salinity gradient (Figure 2B). These rates are similar to those measured in other coastal marine systems (Horak et al., 2013; Damashek et al., 2016; Heiss and Fulweiler, 2016; Laperriere et al., 2018). Undetectable and low nitrification rates in the upstream estuary are likely due to the presence of cyanoHABs during sampling and increased competition for NH_4_^+^. High photoautotrophic NH_4_^+^ uptake rates (2.32 μmol L^–1^ h^–1^; Hampel et al., 2019), coincident with high *Microcystis* cell densities in the upstream estuary (Kramer et al., 2018), suggest that nitrifiers were outcompeted for NH_4_^+^. In contrast, the bloom was less pronounced downstream (Kramer et al., 2018), and photoautotrophic NH_4_^+^ uptake rates were lower (1.11 μmol L^–1^ h^–1^; Hampel et al., 2019), which may help explain the increase in nitrification rates along the salinity gradient.

Before the hurricane, flows of lake water into the estuary were low in 2017, and cyanoHABs were mostly constrained to the lake (Hampel et al., 2019). Nitrification rates in the estuary were an order of magnitude higher in August 2017 than in the previous year (Figure 2B). The highest nitrification rates were observed at the more saline SLE5 and SLE7 (563–978 nmol L^–1^ d^–1^; Figure 2B), and nitrification accounted for 3–7% of total NH_4_^+^ uptake, suggesting a more effective competition for NH_4_^+^. Nitrification rates of this magnitude have been observed in other high-nutrient environments, such as the Eastern Tropical South Pacific (Lipschultz et al., 1990), the hypoxic zone in the Gulf of Mexico (Bristow et al., 2015), coastal Georgia (United States; Tolar et al., 2017), and Hood Canal, Puget Sound (United States; Urakawa et al., 2014). These results illustrate indirect downstream consequences of HABs along the hydrologic continuum, where HABs and nutrient loading upstream in the estuary can suppress nitrification (as seen in 2016), therefore potentially reducing N removal via denitrification.

### Nitrification Following Hurricane Irma

After the hurricane, nitrification rates in both systems increased by an order of magnitude or more (Figure 2). In the lake, nitrification rates were up to 1280 nmol L^–1^ d^–1^ (Figure 2A). Nitrification rates on this scale in natural, freshwater systems have, to our knowledge, only been reported in Lake Taihu (Hampel et al., 2018) and Lake Mendota (Hall, 1986). In the estuary, nitrification rates after the hurricane ranged from 3590–4030 nmol L^–1^ d^–1^, much higher than rates reported previously in oligotrophic and some eutrophic estuarine systems (Horak et al., 2013; Bronk et al., 2014; Urakawa et al., 2014; Heiss and Fulweiler, 2016). Rates in this range were previously measured in the Chang Jiang (Yangtze) River plume (Hsiao et al., 2014), eutrophic Elbe estuary (Sanders et al., 2018), Pearl River estuary (Dai et al., 2008), and northern Gulf of Mexico (Carini et al., 2010). The dramatic increase in nitrification following the hurricane may be related to sediment resuspension caused by high winds, introducing benthic nitrifiers and NH_4_^+^ accumulated in sediments into the water column. With more favorable oxygen conditions in the water column, a fresh supply of NH_4_^+^, and higher turbidity, photoautotrophs may have experienced light limitation (James et al., 2008), thus releasing competitive pressure and allowing nitrification to increase (Wengrove et al., 2015).

A comprehensive review of nitrification in estuaries reported a trend of elevated nitrification rates at Estuary Turbidity Maxima (ETM; Damashek and Francis, 2018), some of which were also associated with turbid waters and strong wind events (Hsiao et al., 2014; Happel et al., 2018; Laperriere et al., 2018; Sanders et al., 2018). NH_4_^+^ released from resuspended sediments can thus lead to nitrification “hot spots” (Percuoco et al., 2015; Damashek et al., 2016). Hurricanes are also accompanied by increased rainfall, leading to larger runoff and increased nutrient concentrations in the water column (Havens et al., 2001; James et al., 2008). High turbidity in the water column may limit photoautotrophic access to light, thus favoring nitrifiers, which are chemolithoautotrophic and do not require light. Microcystin synthetase (*mcyD*) abundance and chl *a* levels decreased following the hurricane (Hampel et al., 2019), supporting this interpretation. In the estuary, nitrification accounted for 60–90% of total NH_4_^+^ uptake after the hurricane, further supporting the idea that turbid conditions favored nitrifiers.

### Ammonia-Oxidizer Community Structure and Abundance

For most sampling events, AOA abundance was greater than AOB (Figure 3). While AOA are generally more abundant than AOB in the open ocean, estuarine ammonia-oxidizing communities are more complex and dynamic, with different systems exhibiting AOA dominance (Beman and Francis, 2006; Horak et al., 2013), AOB dominance (Bernhard et al., 2010), or interchanging spatial dominance of AOA and AOB (Santoro et al., 2008; Zhang et al., 2014). Salinity can help shape ammonia oxidizer community structure (Caffrey et al., 2007; Mosier and Francis, 2008; Santoro et al., 2008). AOB often dominate lower salinity regions of estuaries, while AOA are more abundant in more marine areas (Bouskill et al., 2012), but this pattern is not universal (Mosier and Francis, 2008; Santoro et al., 2008). In the estuary, AOB abundance was only greater than AOA at the upstream, lower salinity site (SLE80).

Despite AOA outnumbering AOB in many systems, AOB abundance has more often correlated positively with nitrification rates (Bernhard et al., 2010; Zeng et al., 2012; Damashek et al., 2015; Hampel et al., 2018). However, the only observed relationship between *amoA* abundance and nitrification rate was between AOA *amoA* copy number and log-transformed nitrification rates in Lake Okeechobee (*R*^2^ = 0.94; Figure 4 and Supplementary Figure S3). A relationship between AOA *amoA* abundance and potential nitrification rates in sediments was reported in hypereutrophic Lake Taihu (Zeng et al., 2012), but we are not aware of any similar relationship reported in a eutrophic lake water column to date.

This study presents a remarkable phylogenetic diversity of recovered AOA OTUs, with close relatives from three of the four major lineages (Alves et al., 2018). Unlike AOB, AOA rarefaction curves (Supplementary Figure S4) did not plateau, suggesting that much of the diversity for AOA was not characterized. Despite the relatively low number of AOA sequences recovered, AOA were still more abundant and diverse than AOB. The low number of sequences, but high diversity and abundance, justifies not rarefying our sequences in the downstream analysis. Recent studies suggest that normalizing sequences via rarefaction can result in discarding important reads and introduces more bias (McMurdie and Holmes, 2014). Overall, the high diversity (even at comparatively low abundance) of AOA sequences along with strong correlation with nitrification rates emphasizes the possible importance of AOA in eutrophic systems and their contribution to nitrification.

The high abundance and diversity of AOA, and strong relationship with nitrification rates within Lake Okeechobee, is puzzling considering the reported oligotrophic nature of AOA (Martens-Habbena et al., 2009; Bollmann et al., 2014; Kits et al., 2017). We suggest that AOA in these eutrophic ecosystems might have evolved from niche differentiation and could represent a new, eutrophic ecotype, separate from oligotrophic AOA (Beman et al., 2008; Sintes et al., 2013; Santoro et al., 2017). High phylogenetic diversity of AOA in this and other studies (Alves et al., 2018; Cheung et al., 2019; Zhao et al., 2020) suggests that broad classification into shallow and deep marine and terrestrial ecotypes is insufficient, and many *amoA* clades represent ecotype intra-diversity, perhaps dictated by other environmental conditions beyond depth, salinity, and pH.

The majority of lake-only AOA OTUs clustered within the NS δ clade, the most abundant clade in the Alves et al. (2018) database that still lacks cultivated representatives. Although little is known about the physiological characteristics of the NS δ clade, it is abundant in soils, freshwater water columns and sediments, marine sediments, wastewater treatment plants, and salt lakes, suggesting an exceptional versatility of habitats for this group. A BLAST search of the representative sequences from this clade matched with previous studies from sludges (Xia et al., 2019), soils (Li et al., 2015; Zhong et al., 2016), and streams affected by wastewater treatment plants (Merbt et al., 2015). Other lake OTUs clustered in NT β and NP δ and η. The NT β clade has no isolated representative and is mostly present in freshwaters, soils, and sediments, while NP δ is also uncharacterized, with sequences from freshwater, estuarine, and marine environments (Alves et al., 2018). These lake-only OTUs, particularly from the NS δ clade at LOBG, could belong to a eutrophic AOA ecotype that has adapted to high NH_4_^+^ concentrations, but evidence from the literature is lacking on AOA communities in lakes. The NH_4_^+^ concentration at this station during sampling was ∼20 μM (Supplementary Table S1), which greatly exceeds typical concentrations in the ocean. This eutrophic AOA ecotype may be able to outcompete AOB in eutrophic freshwater systems, as suggested from our results in Lake Okeechobee. Similar conclusions were reached in the eutrophic Pearl River estuary (Zou et al., 2019), but genomic adaptations of AOA in eutrophic systems and their contributions to nitrification remain an intriguing knowledge gap requiring further investigation.

Few studies have documented changes in ammonia oxidizer community structure following disturbances in aquatic systems. Responses of ammonia oxidizers to various perturbations differ and include increased AOB diversity or abundance (wastewater, Aalto et al., 2018; flow restriction, Bernhard et al., 2015; flooding, Bouskill et al., 2011; storms, Kan, 2018), decreased AOA diversity or abundance (storms, Happel et al., 2018; hurricanes, Newell et al., 2014), or little to no effect (oil spill, Bernhard et al., 2019; oil spill, Marton et al., 2015). Here, we observed substantial hurricane impacts on the abundance, community composition, and diversity of ammonia oxidizers. In the lake, only AOB abundance increased after the hurricane, while in the estuary, we observed the opposite, with AOA abundance increasing. AOB communities were more diverse following the hurricane, while AOA diversity increased in the estuary but decreased in the lake (Figure 8). These results agree with those from a previous study in the Gulf of Mexico (Newell et al., 2014), where benthic AOA communities were less diverse following Hurricanes Ike and Gustav, suggesting a weak resistance to disturbance. Increased AOB abundance in our study also supports disturbance studies in boreal lake sediments (Aalto et al., 2018) and estuaries (Happel et al., 2018), where AOB diversity and/or abundance increased at affected sites, suggesting that AOB are more adaptable to disruptive events (Aalto et al., 2018).

Hurricane disturbance caused a significant shift in AOA community structure in both systems (Figure 5A). In the lake, the community changed from high abundance of low-salinity tolerant NP γ 2.2 (*Nitrosoarchaeum* spp.) to dominance by NP η 1.1 (*Ca. Nitrosotenuis cloacae*) in the central lake (L004, LZ40) and NS δ OTUs at the littoral station (LOBG). *Ca. Nitrosotenuis cloacae* was enriched from a wastewater treatment plant and exhibits intolerance to high salinity, and phylogenetically related strains of NP η have been found in lakes and rivers (Li et al., 2016; Alves et al., 2018). *Nitrososphaera* are often present in soils and sediments (Alves et al., 2018; Li et al., 2018); thus, their high abundance at the littoral station after the hurricane is likely due to sediment resuspension or soil runoff from heavy rainfall. A significant shift in the community was also observed in the estuary, from the salinity tolerant *Nitrosopumilus* spp. (NP γ 2.1) to salinity intolerant *Nitrosoarchaeum* spp. (NP γ 2.2) and *Ca. Nitrosotenuis cloacae* (NP η 1.1), likely reflecting population transfer via water discharges from the lake into the estuary.

In contrast, AOB communities after the hurricane exhibited no community composition changes. The majority of the sequences from both systems were unclassified *Nitrosomonas* and *Nitrosomonas oligotropha* (Figures 5B, 7). A BLAST search of unclassified *Nitrosomonas* consensus sequences revealed that sequences from this study matched with those from other eutrophic systems, such as the Chang Jiang River basin (Jiang et al., 2017), Lake Taihu (Zeng et al., 2012; Zhao et al., 2013; Liu and Yang, 2019), San Francisco Bay (Damashek et al., 2015), an aquaculture system (Bartelme et al., 2017), and wastewater/sludge bioreactors (Wells et al., 2009; Wu et al., 2013; Zhang et al., 2018). Overall, a high abundance of *Nitrosomonas*-like AOB is expected due to NH_4_^+^-rich, eutrophic conditions (Stein et al., 2007; Prosser et al., 2014; Sedlacek et al., 2019) and have been found in many eutrophic natural environments (Zhang et al., 2014; Damashek et al., 2015) and N-rich engineered systems (Zhang et al., 2014; Bartelme et al., 2017). However, some *Nitrosomonas* species are adapted to lower NH_4_^+^ environments. Representative sequences from this study matched *Nitrosomonas sp.* Is79, an ammonia oxidizer found in low NH_4_^+^ freshwater environments (Bollmann et al., 2013). Additionally, sequences in this study classified as *Nitrosomonas oligotropha*, which is also adapted to lower trophic conditions (French et al., 2012). In the estuary, we observed an increase in *Nitrosomonas oligotropha* after the hurricane, possibly due to reduced salinity (Mosier and Francis, 2008), sediment resuspension, and lower nutrient concentrations (NH_4_^+^ and ortho-P; Supplementary Table S2).

The increased nitrifier abundance, shift in AOA community structure, and higher nitrification rates post-hurricane were likely caused by a combination of physicochemical factors, resuspension of benthic nitrifiers into the water column, and reduced competition with cyanobacteria. Environmental parameters explaining variance in nitrifier community structure were salinity, turbidity, and NH_4_^+^ and NO_3_^–^ concentrations (Supplementary Figure S2). All of these factors were affected by the hurricane disturbance (Supplementary Tables S1, S2). The presence of primarily soil and sediment nitrifiers in water column samples likely contributed to increased nitrification rates, along with reduced competition with *Microcystis*. The shift (PERMANOVA; *p* = 0.003) in AOA community structure, but not AOB, might indicate that AOA have lower resistance to disturbance than AOB (Bowen et al., 2011; Shade et al., 2011, 2012), but a follow-up study would be required to assess resilience.

## Conclusion

This study evaluated the effects of cyanoHABs and Hurricane Irma on water column nitrification rates and ammonia oxidizer community structure along a freshwater-estuarine continuum. We show that nitrification rates were lower during cyanoHABs, increasing by an order of magnitude when cyanoHABs were less abundant in the estuary the following year. We also show that several OTUs from Lake Okeechobee belong to the uncultured NS δ clade, which is versatile and appears to be ecologically important in eutrophic systems. Following Hurricane Irma, nitrification rates increased by an order of magnitude or more, coincident with increased turbidity and conductivity and decreased salinity and cyanoHABs. We also observed a substantial shift in AOA community structure immediately after the hurricane, suggesting weaker resistance to disturbance than AOB.

This study contributes to the growing literature showing high abundance of AOA in eutrophic waters relative to AOB and presents a strong relationship between AOA abundance and nitrification rates. Given their relatively recent discovery and large gaps in knowledge of AOA physiology, many open questions and suggestions for future research remain:

(1) AOA appear to be adapted to a wider range of trophic conditions than previously thought. The high abundance and diversity of AOA in eutrophic systems, and low number of cultured species (NS δ, NT β, NP δ), suggest that trophic adaptation and niche differentiation are likely, beyond the oceanic/terrestrial ecotype differentiation;
(2) AOB alpha diversity increased after the hurricane, while the AOA community shifted dominance. However, it is not certain which of these groups was responsible for the increase in nitrification rates in the estuary after the hurricane;
(3) Our results present short-term effects of hurricane and cyanoHAB disturbances on nitrification rates and ammonia oxidizer community structure. Studies focused on longer term effects should explore resilience of the ammonia oxidizer community and temporal scales of community recovery. The limited scope of the present study leaves open whether and/or how quickly the community might return to the pre-hurricane state, when turbidity is either flushed or settles to the sediment surface. Current climate change projections forecast amplification of hurricane events and increased eutrophication, and understanding the role of nitrification in converting chemically reduced N forms to oxidized N, and providing substrate for N removal via denitrification, may present valuable opportunities to constrain system N budgets.

## Data Availability Statement

The datasets presented in this study can be found in online repositories. The names of the repository/repositories and accession number(s) can be found in the article/ Supplementary Material.

## Author Contributions

JH, MM, and SN designed the study and collected samples. JH analyzed the samples. SA helped with sequence analysis. JH, SN, and MM interpreted results. All authors contributed to writing and revising the manuscript.

## Conflict of Interest

The authors declare that the research was conducted in the absence of any commercial or financial relationships that could be construed as a potential conflict of interest.

## Footnotes

1 https://www.sfwmd.gov/science-data/dbhydro
