# Supplementary material for: Hurricane Disturbance Stimulated Nitrification and Altered Ammonia Oxidizer Community Structure in Lake Okeechobee and St. Lucie Estuary (Florida)
Source: Front Microbiol. 2020 Jul 10;11:1541. doi: 10.3389/fmicb.2020.01541 (PMC7366250; doi:10.3389/fmicb.2020.01541)
Supplement: Supplementary file 1 [file Table_1.DOCX]

Supplementary Information

**Hurricane disturbance stimulated nitrification and altered ammonia oxidizer community structure in Lake Okeechobee and St. Lucie Estuary (Florida).**

Justyna J. Hampel, Mark J. McCarthy, Sanni L. Aalto, Silvia E. Newell

Figure S1. Results of Non-metric multidimensional scaling analysis of *amoA* sequences in Lake Okeechobee and St. Lucie Estuary.

(a) Ammonia Oxidizing Archaea


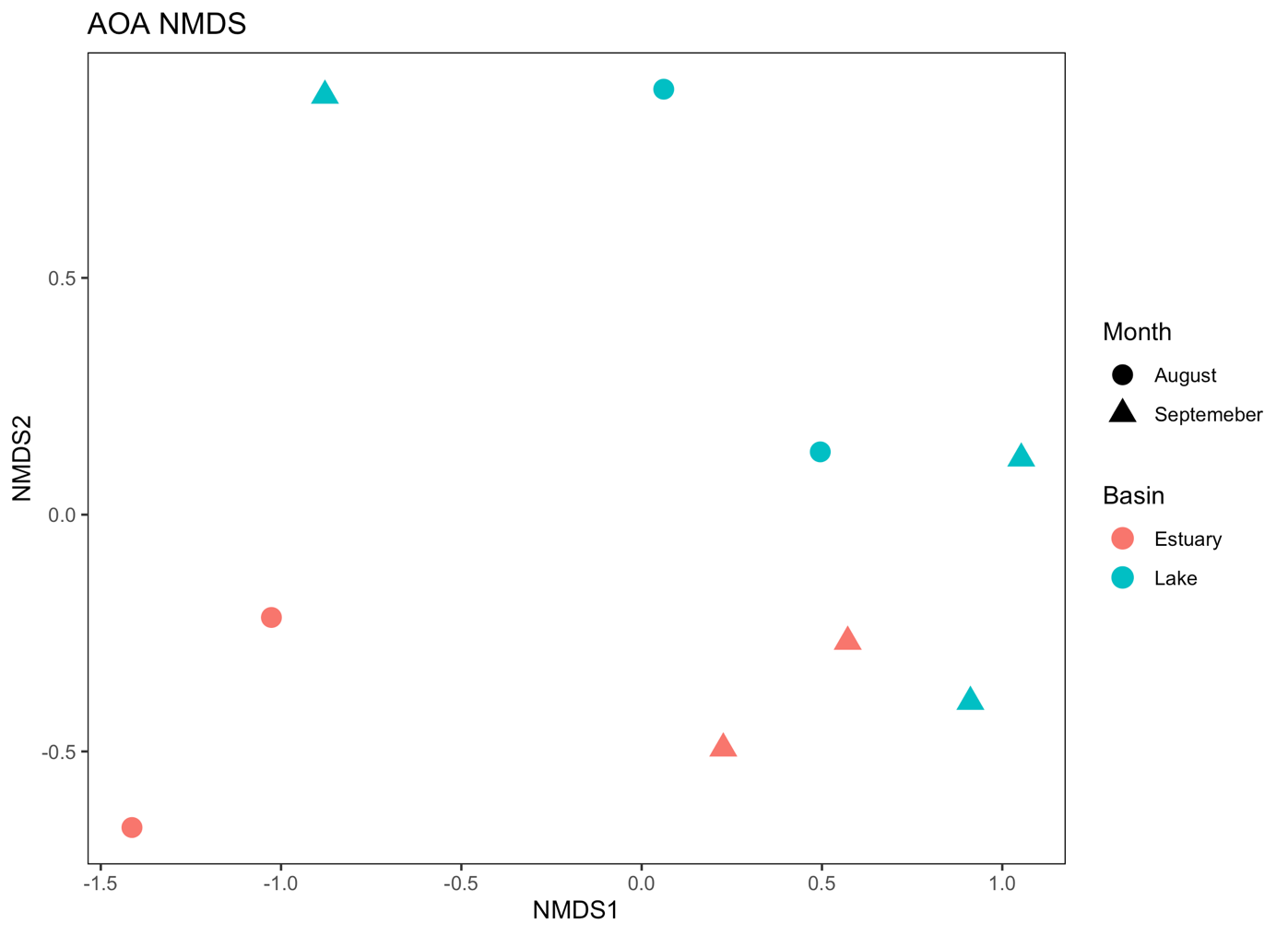


Stress: 0.07434207

(b) Ammonia Oxidizing Bacteria


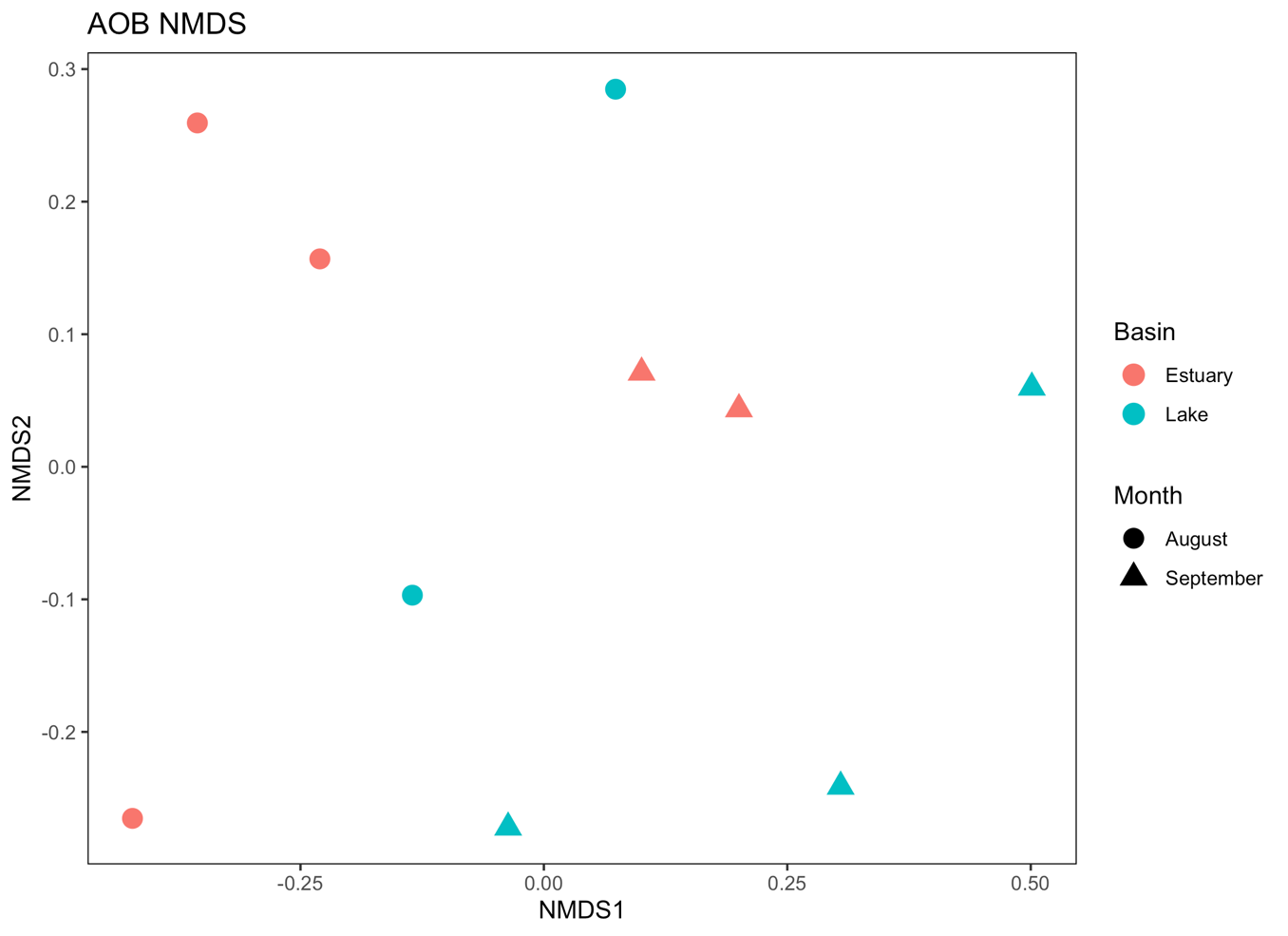


Stress: 0.1361871

Figure S2. Results of Canonical Analysis of Principal coordinates between ammonia oxidizers, nitrification rates (NTR), and environmental variables: dissolved oxygen (DO), salinity, turbidity, ammonium (NH4) and nitrate (NO3).

(a) Ammonia Oxidizing Archaea


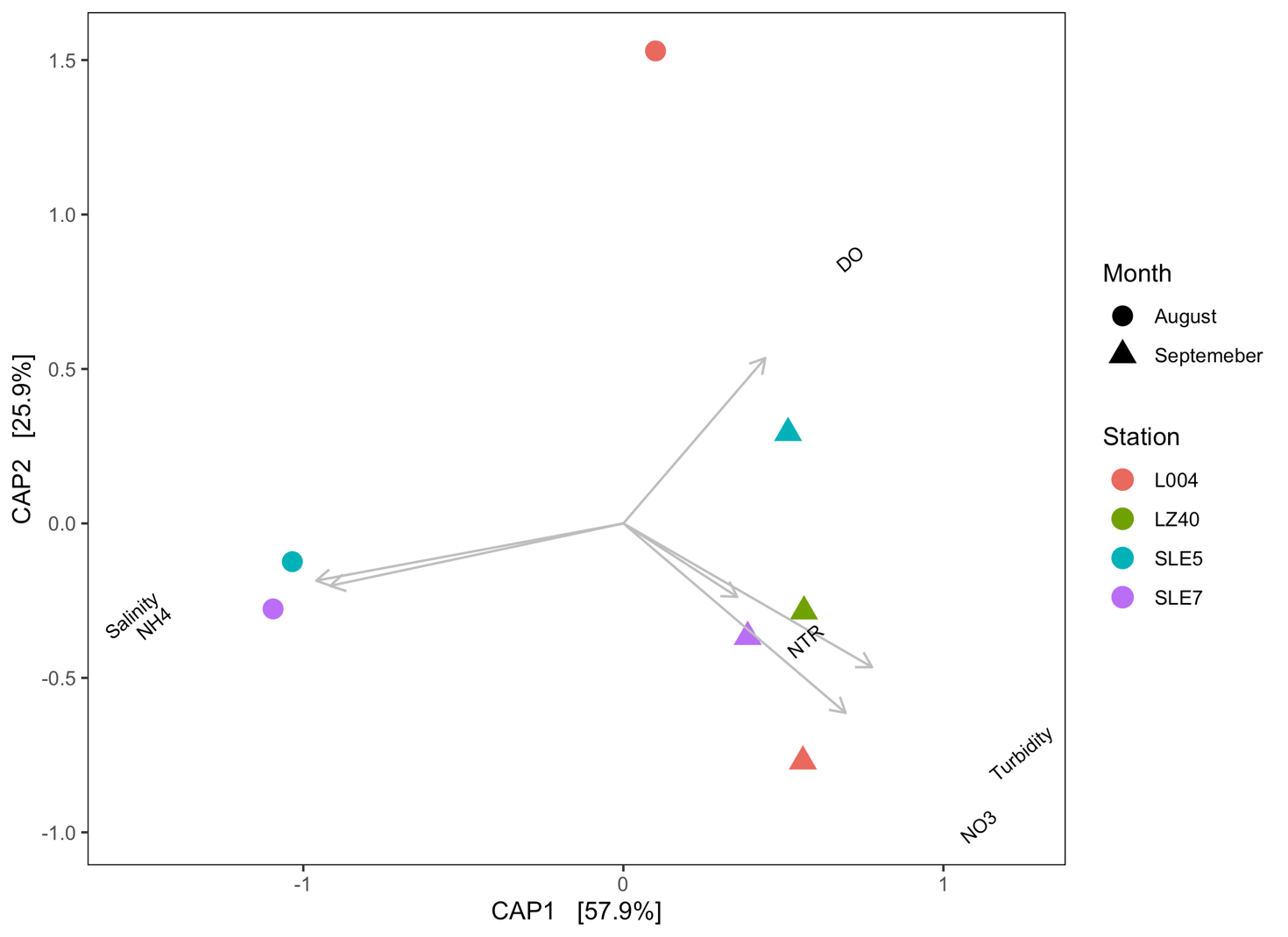


(b) Ammonia Oxidizing Bacteria


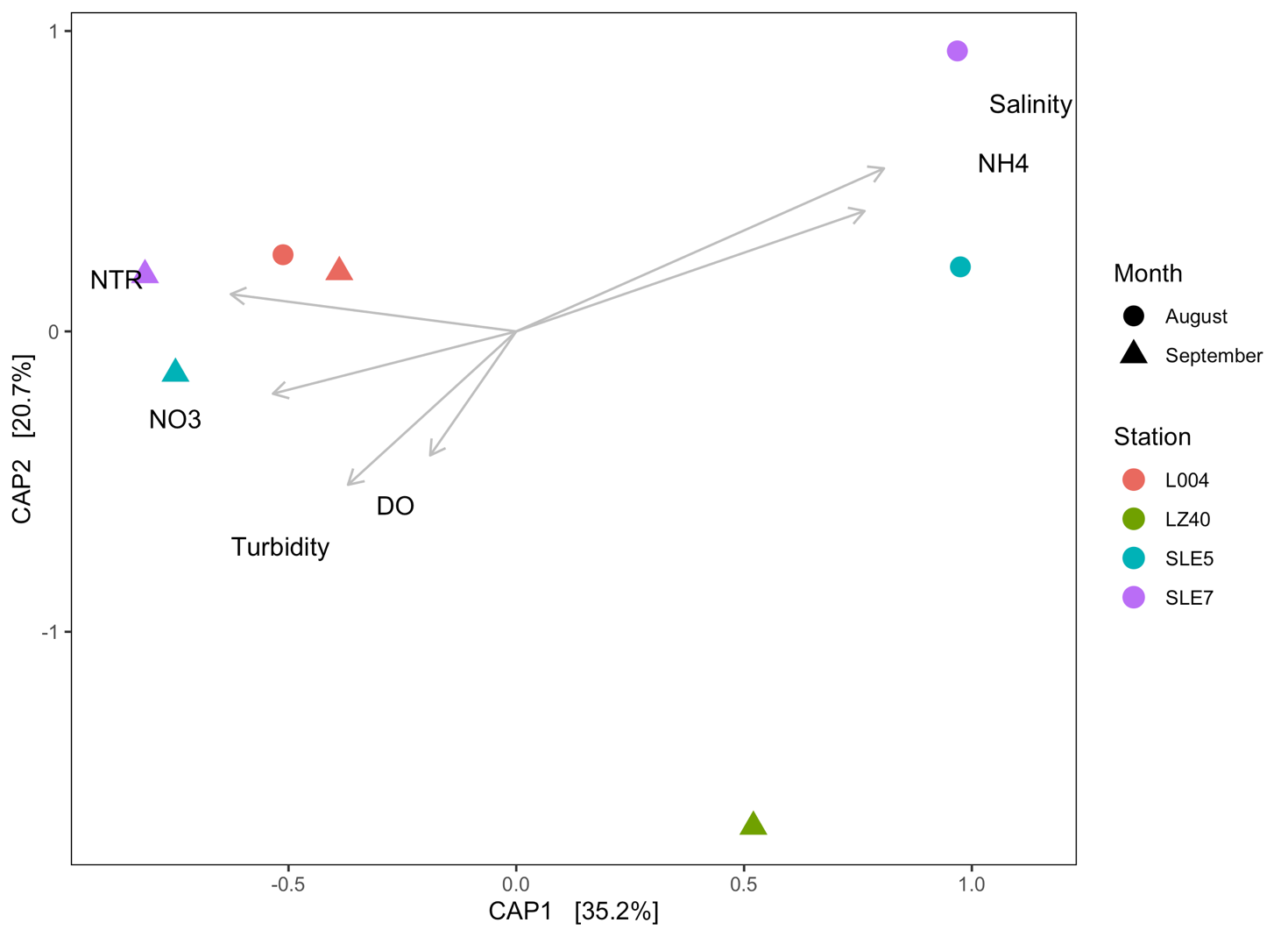


Figure S3. Pearson correlation between log-transformed nitrification rates and (A) ammonia oxidizing archaea in Lake Okeechobee, (B) ammonia oxidizing bacteria in Lake Okeechobee, (C) ammonia oxidizing archaea in St. Lucie Estuary, and (D) ammonia oxidizing bacteria in St. Lucie Estuary.

Figure S4. Rarefaction curves

(a) Ammonia Oxidizing Archaea


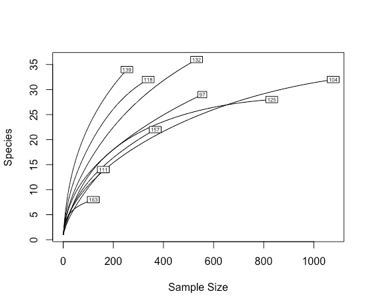


(b) Ammonia Oxidizing Bacteria


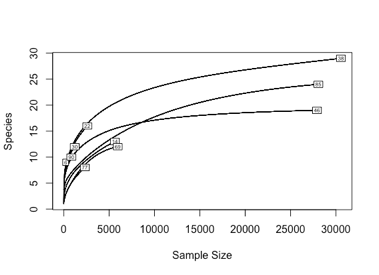


| Table S1. Environmental data and ambient nutrient concentrations in Lake Okeechobee in 2016 and 2017. Temperature, dissolved oxygen (DO), pH, chlorophyll a (Chla), and conductivity (Cond.) were only measured in surface waters. Nutrient concentrations (ammonium (NH_4_^+^), nitrite (NO_2_^-^), nitrate (NO_3_^-^), orthophosphate (OP), and urea) were measured in triplicate within ± 10% error margin. Total Nitrogen (TN), Total Phosphorus (TP), turbidity and total suspended solids (TSS) in Lake Okeechobee were obtained from DB Hydro database (South Florida Water Management District). | | | | | | | | | | | | | | | | |  |  |  |  |  |  |  |
| --- | --- | --- | --- | --- | --- | --- | --- | --- | --- | --- | --- | --- | --- | --- | --- | --- | --- | --- | --- | --- | --- | --- | --- |
| Sampling Date | Station | Temp | DO | pH | Chla | Cond. | NH_4_^+^ | NO_2_^-^ | NO_3_^-^ | OP | Urea | TN | TP | Turbidity | TSS |  |  |  |  |  |  |  |  |
|  |  | °C | mg/L |  | µg/L | µs/cm | µM | µM | µM | µM | µM | µM | µM | NTU | mg/L |  |  |  |  |  |  |  |  |
| 27 Jul 2016 | LO04 | 30.9 | 11.9 | 8.09 | 27.1 | 360 | 6.70 | 0.09 | 0.11 | 1.10 | 2.46 | 68.4 | 3.35 |  |  |  |  |  |  |  |  |  |  |
|  | LO04 D |  |  |  |  |  | 7.75 | 0.29 | 1.20 | 0.96 | 2.56 |  |  |  |  |  |  |  |  |  |  |  |  |
|  | LZ40 | 31.5 | 9.81 | 7.94 | 25.3 | 383 | 0.11 | 0.21 | 1.18 | 0.98 | 1.28 | 131 | 4.90 |  |  |  |  |  |  |  |  |  |  |
|  | LZ40 D |  |  |  |  |  | 0.82 | 0.21 | 1.56 | 1.02 | 3.70 |  |  |  |  |  |  |  |  |  |  |  |  |
| 22 Aug 2017 | LO04 | 29.6 | 8.24 | 8.17 | 33.5 | 372 | 0.19 | 0.07 | 2.06 | 0.94 | 2.44 | 103 | 4.39 | 23.7 | 25 |  |  |  |  |  |  |  |  |
|  | LO04 D |  |  |  |  |  | 0.11 | 0.07 | 1.74 | 0.96 | 0.93 |  |  |  |  |  |  |  |  |  |  |  |  |
|  | SAV 165 | 29.8 | 10.2 | 8.95 | 77.8 | 368 | 0.59 | 0.03 | 0.00 | 0.07 | 1.10 | 114 | 1.74 |  |  |  |  |  |  |  |  |  |  |
| 22 Sept 2017 | LO04 | 28.4 | 6.47 | 7.91 | 11.3 | 391 | 0.11 | 0.08 | 53.6 | 1.05 | 1.90 | 164 | 7.42 | 114 | 81 |  |  |  |  |  |  |  |  |
|  | LZ40 | 28.1 | 7.63 | 8.16 | 8.16 | 405 | 0.58 | 0.10 | 24.4 | 0.75 | 3.41 | 117 | 5.19 | 82 | 49 |  |  |  |  |  |  |  |  |
|  | LOBG | 29.8 | 2.47 | 7.36 | 22.0 | 556 | 19.1 | 3.28 | 18.7 | 4.93 | 4.34 |  |  |  |  |  |  |  |  |  |  |  |  |

| Table S2. Environmental data and ambient nutrient concentrations in St. Lucie Estuary in 2016 and 2017. Temperature, dissolved oxygen (DO), pH, chlorophyll a (Chla), salinity (Sal.), and conductivity (Cond.) were measured with a YSI sonde. Ammonium (NH_4_^+^), nitrite (NO_2_^-^), nitrate (NO_3_^-^), orthophosphate (OP), and urea were measured in triplicate within ± 10% error margin. Total Nitrogen (TN), Total Phosphorus (TP), turbidity and total suspended solids (TSS) in St. Lucie Estuary were obtained from DB Hydro database (South Florida Water Management District). | | | | | | | | | | | | | | | | | |  |  |  |  |  |  |  |  |  |
| --- | --- | --- | --- | --- | --- | --- | --- | --- | --- | --- | --- | --- | --- | --- | --- | --- | --- | --- | --- | --- | --- | --- | --- | --- | --- | --- |
| Sampling Date | Station | Temp | DO | pH | Chla | Sal. | Cond. | NH_4_^+^ | NO_2_^-^ | NO_3_^-^ | OP | Urea | TN | TP | Turbidity | TSS |  |  |  |  |  |  |  |  |  |  |
|  |  | °C | mg/L |  | µg/L |  | µs/cm | µM | µM | µM | µM | µM | µM | µM | NTU | mg/L |  |  |  |  |  |  |  |  |  |  |
| 25 Jul 2016 | SLE80 | 31.1 | 7.1 | 7.4 | 12.1 | 0.19 | 0.395 | 3.19 | 0.82 | 6.67 | 2.03 | 3.22 | 80.0 | 3.87 |  |  |  |  |  |  |  |  |  |  |  |  |
|  | SLE2 | 30.2 | 7.4 | 7.5 | 19.3 | 0.19 | 0.399 | 1.87 | 0.77 | 7.09 | 1.46 | 1.05 |  |  |  |  |  |  |  |  |  |  |  |  |  |  |
|  | SLE4 | 29.6 | 7.6 | 7.6 | 8.9 | 0.2 | 0.42 | 3.61 | 1.06 | 7.59 | 1.30 | 1.86 | 82.9 | 5.16 |  |  |  |  |  |  |  |  |  |  |  |  |
|  | SLE8 | 29.8 | 6 | 7.6 | 10.2 | 8.9 | 15.5 | 2.95 | 0.42 | 3.00 | 3.28 | 1.37 | 58.6 | 5.16 |  |  |  |  |  |  |  |  |  |  |  |  |
| 24 Aug 2017 | SLE80 | 29.6 | 5.9 | 7.9 | 21.1 | 0.33 | 0.686 | 7.83 | 0.81 | 10.4 | 4.46 | 5.47 | 78.6 | 5.16 |  |  |  |  |  |  |  |  |  |  |  |  |
|  | SLE5 | 29.7 | 4.2 | 7.4 | 16.1 | 4.64 | 8.38 | 12.2 | 2.90 | 9.17 | 6.43 | 4.00 | 91.4 | 9.03 | 9.7 | 12 |  |  |  |  |  |  |  |  |  |  |
|  | SLE7 | 29.4 | 5.6 | 7.7 | 16.8 | 6.75 | 11.7 | 7.39 | 5.68 | 7.49 | 6.66 | 3.31 | 81.4 | 8.06 | 7.2 | 9 |  |  |  |  |  |  |  |  |  |  |
| 21 Sept 2017 | SLE5 | 29.4 | 6.9 | 7.5 | 13.6 | 0.19 | 405 | 0.25 | 0.34 | 34.6 | 2.48 | 2.04 | 124 | 6.77 | 45.9 | 30 |  |  |  |  |  |  |  |  |  |  |
|  | SLE7 | 29.4 | 3.9 | 7.5 | 12.8 | 0.22 | 466 | 2.22 | 1.09 | 35.3 | 4.45 | 5.13 | 127 | 6.77 | 46 | 22 |  |  |  |  |  |  |  |  |  |  |

| Table S3. Details of best-fitting multiple regression models determined by stepwise regression for nitrification rates and environmental variables in Lake Okeechobee and St. Lucie Estuary. All variables were log-transformed prior to analysis. | | | | | | | | | | | | | | | | | | | |  |
| --- | --- | --- | --- | --- | --- | --- | --- | --- | --- | --- | --- | --- | --- | --- | --- | --- | --- | --- | --- | --- |
| Process | | Variable | |  | | Parameter | | |  | |  | | Model | |  | | |  |  | |
|  | |  | | Estimate | | Std. estimate | | | P | | Adj. R2 | | F | | P | | |  |  | |
| Nitrification | | NO_3_^-^ | | 3.630 | | 0.190 | | | 0.002 | | 0.994 | | 283 | | 0.003 | | |  |  | |
|  | | Salinity | | -0.957 | | 0.250 | | | 0.063 | |  | |  | |  | | |  |  | |
|  | | Turbidity | | -3.012 | | 0.330 | | | 0.012 | |  | |  | |  | | |  |  | |
|  | |  | |  | |  | | |  | |  | |  | |  | | |  |  | |
|  | |  | |  | |  | | |  | |  | |  | |  | | |  |  | |
|  | |  | |  | |  | |  |  | |  | |  | |  | |  |  |  |  |

Table S4. Details of the amoA abundance and sequencing analyses.

| qPCR and sequencing Step 1 |  |  |  |  |
| --- | --- | --- | --- | --- |
| Name | Original Sequence (5' to 3') | qPCR Program | PCR program | Reference |
| Arch-amoAF | STAATGGTCTGGCTTAGACG | 95℃ for 2 minutes; 40 cycles of 95℃ for 30 seconds, 53℃ for 45 seconds and 72℃ for 1 min; final extensions of 72℃ for 5 minutes | 95℃ for 5 minutes; 24 cycles of 95℃ for 30 seconds, 53℃ for 45 seconds and 72℃ for 1 min; final extensions of 72℃ for 5 minutes | Francis et al., 2005  Bollmann et al., 2008 |
| Arch-amoAR | GCGGCCATCCATCTGTATGT |  |  |  |
| AOB-amoAF | GGGGTTTCTACTGGTGGT | 95℃ for 2 minutes; 40 cycles of 94℃ for 45 seconds, 56℃ for 30 seconds and 72℃ for 1 min; final extensions of 72℃ for 5 minutes | 95℃ for 5 minutes; 24 cycles of 94℃ for 45 seconds, 56℃ for 30 seconds and 72℃ for 1 min; final extensions of 72℃ for 5 minutes | Rotthauwe 1997 |
| AOB-amoAR | CCCCTCKGSAAAGCCTTCTTC |  |  |  |
|  |  |  |  |  |
| Sequencing Step 2 (Barcoded primers) | Original Sequence (5' to 3') |  | PCR program | Reference |
| Arch-amoAF | TCGTCGGCAGCGTCAGATGTGTATAAGAGACAGSTAATGGTCTGGCTTAGACG |  | Like Step 1. 8 cycles | Herebold et al., 2015 |
| Arch-amoAR | GTCTCGTGGGCTCGGAGATGTGTATAAGAGACAGGCGGCCATCCATCTGTATGT |  |  |  |
| AOB-amoAF | TCGTCGGCAGCGTCAGATGTGTATAAGAGACAGGGGGTTTCTACTGGTGGT |  | Like Step 1. 8 cycles |  |
| AOB-amoAR | GTCTCGTGGGCTCGGAGATGTGTATAAGAGACAGCCCCTCKGSAAAGCCTTCTTC |  |  |  |
